# N-Acetyl-L-Cysteine Prevents Stress-Induced Desmin Aggregation in Cellular Models of Desminopathy

**DOI:** 10.1371/journal.pone.0076361

**Published:** 2013-10-01

**Authors:** Bertrand-David Segard, Florence Delort, Virginie Bailleux, Stéphanie Simon, Emilie Leccia, Blandine Gausseres, Fatma Briki, Patrick Vicart, Sabrina Batonnet-Pichon

**Affiliations:** 1 University Paris Diderot, Sorbonne Paris Cité, Unité de Biologie Fonctionnelle et Adaptative, CNRS EAC4413, Paris, France; 2 University Paris Sud, Paris 11, Laboratoire de Physique des solides, Orsay, France; UMCG, Netherlands

## Abstract

Mutations within the human *desmin* gene are responsible for a subcategory of myofibrillar myopathies called desminopathies. However, a single inherited mutation can produce different phenotypes within a family, suggesting that environmental factors influence disease states. Although several mouse models have been used to investigate organ-specific desminopathies, a more general mechanistic perspective is required to advance our knowledge toward patient treatment. To improve our understanding of disease pathology, we have developed cellular models to observe desmin behaviour in early stages of disease pathology, e.g., upon formation of cytoplasmic desmin aggregates, within an isogenic background. We cloned the wildtype and three mutant desmin cDNAs using a Tet-On Advanced® expression system in C2C12 cells. Mutations were selected based on positioning within desmin and capacity to form aggregates in transient experiments, as follows: DesS46Y (head domain; low aggregation), DesD399Y (central rod domain; high aggregation), and DesS460I (tail domain; moderate aggregation). Introduction of these proteins into a C2C12 background permitted us to compare between desmin variants as well as to determine the role of external stress on aggregation. Three different types of stress, likely encountered during muscle activity, were introduced to the cell models—thermal (heat shock), redox-associated (H_2_O_2_ and cadmium chloride), and mechanical (stretching) stresses—after which aggregation was measured. Cells containing variant DesD399Y were more sensitive to stress, leading to marked cytoplasmic perinuclear aggregations. We then evaluated the capacity of biochemical compounds to prevent this aggregation, applying dexamethasone (an inducer of heat shock proteins), fisetin or N-acetyl-L-cysteine (antioxidants) before stress induction. Interestingly, N-acetyl-L-cysteine pre-treatment prevented DesD399Y aggregation during most stress. N-acetyl-L-cysteine has recently been described as a promising antioxidant in myopathies linked to selenoprotein N or ryanodin receptor defects. Our findings indicate that this drug warrants further study in animal models to speed its potential development as a therapy for DesD399Y-linked desminopathies.

## Introduction

Mutations in the gene encoding desmin (*DES*, HGNC: 2770) are responsible for the most studied subgroup of myofibrillar myopathies (MFM), diseases affecting cardiac and skeletal muscles, called desminopathies [1]. Desminopathies exhibit characteristic histology, with desmin-containing protein aggregates in cardiac and skeletal muscle cell cytoplasm. Desmin, a type III intermediate filament (IF) protein, is specifically expressed in muscle. This protein forms a cytoplasmic scaffold interconnecting myofibres and linking the contractile apparatus both to the nucleus and to desmosomal proteins [2] in cardiac cells [3] or to costamers in skeletal muscle [4,5]. Generally, desminopathies manifest between the second and fourth decade of life with extreme heterogeneity. Some individuals exhibit progressive skeletal myopathy without cardiac involvement [6]; others present with cardiomyopathy as the first [7–9], or even the only, feature [10].

To date, about sixty different pathological mutations have been identified in *DES* [11]. Most are missense mutations affecting the 2B region of the protein rod domain and leading to desmin network disorganization [12]. Point mutations located in the C-terminal domain are mainly associated with cardiac phenotypes but some of them can also lead to skeletal phenotypes as S460I mutation [5]. A few mutations affecting the protein N-terminal domain are associated with both cardiac and skeletal phenotypes [13]. Interestingly, a single, inherited *DES* mutation can lead to different clinical phenotypes within a single family, suggesting that other genetic and/or environmental factors influence these phenotypes.

Desmin function has been well characterized using knock-out (KO) mouse models. Complete absence of desmin (*Des* ^*-/-*^) produces viable and fertile mice having normal muscle development [14,15]. However, desmin KO causes cell architecture defects and unusual myosin distribution in early stages of mouse development [16]. Additionally, adult KO mice are sensitive to mechanical stress, which increases the severity of the cardiac phenotype and leads to calcification and fibrosis [17]. Further, *Des* ^*-/-*^ muscle cell mitochondria exhibit an increase in size and number, a loss of correct positioning, and pronounced degeneration after exercise overload [18,19].

Some studies investigated how desmin mutations affect both the assembly of the recombinant protein *in vitro* as well as the filament-forming capacity in cDNA-transfected cells [20]. Several mutants assemble *in vitro* into seemingly normal IFs (i.e. DesS460I, DesE245D), despite some presents irregular diameters (DesD399Y, DesS46Y) [20], [11]. Other mutants interfere with the assembly process at distinct stages, i.e., tetramer formation, unit-length filament (ULF) formation, filament elongation, and IF maturation [20]. More recently, *in vitro* studies have identified mechanical properties of wild-type and pathogenic desmin filaments using atomic force microscopy [21,22]. These studies revealed that desmin variants differ in nanomechanical behaviours: DesA360P exhibits tensile properties identical to the wild-type, but DesQ389P and DesD399Y introduce local variations along the filament. These variations alter the biophysical behaviour of desmin polymers, impairing their mechano-sensing and mechano-transduction abilities. Indeed, primary myoblasts expressing the DesR350P variant are stiffer and more prone to stretch-induced injury, leading to abnormal protein aggregation [23].

Moreover transient transfection experiments show different aggregative behaviours depending on cellular context for some mutants. For instance desS460I leads to aggregate formation in SW13 cells, whereas it forms normal filaments in C2C12 [9]. So aggregation depends first on mutant characteristics but also on potential worsening factors as intracellular or environmental context.

Other studies have demonstrated increased levels of the glycoxidation markers AGE, CML, and CEL in muscle samples from individuals with desminopathies, and desmin has been identified as a major target of oxidation and nitration [24]. Taken together, these results suggest that environmental conditions and lifestyle can modulate the severity of the disease.

Although KO mouse models have been instrumental in characterising desmin function, only a few models expressing desmin mutants have been described. Studies of mice expressing DesI451M specifically in cardiac tissue have implicated this desmin mutant in cardiomyopathy onset [25]. Mice expressing DesL345P show marked differences in mitochondrial structure and function in both heart and skeletal muscle cells [26]. Over-expressing Des-delR173-E179 in mouse recapitulates aspects of human desminopathies, specifically intra-sarcoplasmic aggregates and cardiac hypertrophy [27]. Despite their usefulness as models of human disease, mouse studies have not yet extended to testing therapeutic options, except for one HSPB5-R120G cardiac MFM model without desmin primary defect [28], an important avenue to pursue given that no treatment is presently available for desminopathies.

To begin identifying potential treatments for desminopathy, four inducible and stable myoblastic cell models expressing either wild-type (WT) or desmin mutated in N-ter (DesS46Y), 2B rod (DesD399Y), or C-ter (DesS460I) domains were constructed. Desmin aggregation was assessed by immunohistochemistry following application of four distinct stresses (mechanical, thermal, and two redox). We observed significant aggregate formation in the DesD399Y mutant cell line, which had high sensitivity to all stresses. In contrast, DesS46Y and DesS460I mutants were similar to DesWT cells, with little desmin aggregation. Pre-treatment of DesD399Y cells with N-acetyl-L-cysteine (NAC) protected them from desmin aggregation following stress. These findings suggest a novel potential therapeutic approach to treat a subset of desminopathies.

## Materials and Methods

### Plasmids

For transient expression, previously generated human *desmin* wild-type or mutated cDNAs [12] were subcloned in *Eco*RI-*Bam*HI restriction sites of pLink expression vector (containing a 5’ Myc tag). For stable expression, pTRE-Tight (Clontech) plasmid was digested by *Pvu*I-*Eco*RV enzymes to obtain a tetracycline-inducible promoter insert, which was then cloned into a pcDNA_3_-puromycin plasmid. 5’-Myc-tagged complete human *desmin* wild-type or mutated cDNA were then subcloned in this new vector in the *Eco*RI-*Xba*I sites. These constructs were named pPuro-Myc-Des. All plasmids were sequenced (Eurofins, MWG).

### Cell lines, DNA transfection, and culture

Transfection of pLink-desmin constructs in C2C12 cells (ATCC) was performed using the JetPEI method (Ozyme) according to the manufacturer’s instructions. A Tet-On cell line was generated after transfection with pTet-On-advanced (Clontech) containing rtTA-advanced trans-activator (doxycycline sensitive). G418 sulfate (Euromedex, 1 mg/mL for one week) and proliferation selections yielded thirty clones. Doxycycline (Sigma-Aldrich) response was tested using transient transfection with pTet-On-Luc activation control vector (Clontech). Clone 21 was chosen based on proliferation and differentiation abilities.

Double-stable cells expressing WT or mutant (DesS46Y, DesD399Y, DesS460I) desmin were then generated after transfection of clone 21 cells with pPuro-Myc-Des constructs. Subsequently, puromycin (Euromedex, 2 µg/mL) selection was applied for one week. One clone containing an empty vector, named cl 21V, was used as control. Doxycycline induction of human desmin variants was verified by Western blotting (antibody against c-Myc epitope) and immunostaining.

C2C12 cells were grown in Dulbecco’s modified Eagle’s medium (DMEM, Life Technologies) supplemented with 10% foetal bovine serum (PAA) and 1% penicillin/streptomycin (Life Technologies). Stable clones were grown in DMEM, 20% foetal bovine serum, 1% penicillin/streptomycin, 1 mg/mL G418, and 2 µg/mL puromycin.

### Stress procedures

#### Chemical and heat stresses

Cells were seeded at 3 x 10^3^ cells/cm^2^ and induced with doxycycline (10 µg/mL) 24 h later. Chemical and heat stresses were introduced 48 h after induction, as follows: heat shock was performed at 42°C for 2 h; H_2_O_2_ (0.2 mM; Sigma-Aldrich) was added to the cell culture for 2 h; or cadmium chloride (20 µM; Sigma-Aldrich) was added to culture for 6 h. Subsequently, cells were either immediately (T_0_) used for FACS, Western blotting, or immunostaining, or the media was changed and analyzed 24 h later (T_24_).

Anti-aggregative compounds were tested by pre-treating cells for 16 h before stress with either dexamethasone (2 µM), fisetin (10 µM), or NAC (10 mM) (all from Sigma-Aldrich).

#### Cell stretching: device, conditions, and experiments

Cells were stretched as described previously [29]. Briefly, cells were plated on elastomeric strips of poly-dimethylsiloxane (PDMS) (Sylgard 184, Corning) coated with fibronectin (Sigma-Aldrich) at 2 x 10^3^ cells/cm^2^ and cultured for 72 h before stretching. Physiological conditions were maintained during stretching by placing the device in an incubator at 37°C and 5% carbon dioxide.

PDMS substrates were cyclically stretched at 0.3 Hz with maximal strain amplitude of 20%. This frequency allows observation of aggregation in cells after the 6-h experimental time frame, with or without recovery. As a control, cells on PDMS strips were kept at rest and in the same culture conditions as the stretched ones. Four substrates with either WT or mutated desmin-containing cells were stretched together for each experiment, promoting similar cell confluence and passage number.

### Immunohistochemistry

Primary antibodies: (1) mouse monoclonal anti-c-Myc antibody (Santa Cruz Biotechnologies; 1/100); (2) rabbit polyclonal anti-c-Myc (Sigma-Aldrich; 1/1000); (3) mouse monoclonal anti-alpha-tubulin (Molecular Probes, 1/100); (4) rabbit anti-B1 lamin [30], (5) rabbit anti-A/C lamin [31]; (6) anti-calnexin (ER stress kit from Cell Signalling, 1/50). Isotype-specific secondary antibodies with anti-mouse and anti-rabbit Alexa-568 or -488 (Molecular Probes) were used. Actin was detected with Alexafluor 488® phalloidin. For immunofluorescence, cells were fixed with 2% paraformaldehyde for 15 min at room temperature, permeabilized 5 min with 0.5% Triton X-100, then incubated with the primary antibody for 1 h at room temperature. Binding of primary antibodies was detected by incubating cells 40 min with suitable secondary antibodies. DNA was stained with Hoechst (1 µg/mL, Sigma-Aldrich) for 10 min. Finally cells were washed in PBS and mounted in Fluoromount medium (Interchim). Staining was visualized with confocal microscopy (ZEISS LSM700).

### Western blotting

Proteins were extracted using RIPA buffer without SDS, separated by SDS-PAGE, and transferred to nitrocellulose membranes (Macherey Nagel), which were then incubated with 5% non-fat milk in PBS-0.5% Tween. Primary antibody was added at the appropriate dilution and membranes were incubated from 1 h at room temperature to 16 h at 4°C. Primary antibodies used were: (1) mouse monoclonal anti-c-Myc antibody (Santa Cruz Biotechnologies, 1/1000); (2) rabbit polyclonal anti-desmin (Biogenesis, England, 1/250); (3) mouse monoclonal anti-alpha-actin (Millipore, 1/2000); (4) rabbit polyclonal anti-alphaB-crystallin (Stressgen, 1/1000); (5) rabbit polyclonal anti-HSP25 (Stressgen, 1/1000); (6) BIP (ER stress kit from Cell Signalling, 1/1000); (7) CHOP (ER stress kit from Cell Signalling, 1/500). Isotype-specific secondary antibody coupled with a horseradish peroxidase (Pierce, 1/10,000) was detected by incubating with ECL+ (GE Healthcare) and visualized with CCD camera (FUJI Las 4000).

### Counting and Statistics

Images are tile scan 5x5 on 3 random nuclear fields chosen on Hoechst-stained areas. Total cell number was calculated by counting nuclei with an ImageJ software in-house macro. Cells presenting aggregates were visually counted on the images. The ratio between stressed versus unstressed aggregated cells was then calculated. Experiment was repeated at least 3 times. Statistical analysis of the results was realized with non-parametric tests and R software. Significant differences were considered if p < 0.05.

### Flow cytometry assay for ROS quantification

CellROX® Green Reagent (Life Technologies) is a fluorogenic probe for measuring oxidative stress in living cells. The cell-permeable dye is weakly fluorescent while in a reduced state and exhibits bright green photostable fluorescence upon oxidation by reactive oxygen species (ROS). Cells were seeded on 6-well plates at 3 x 10^3^ cells/cm^2^. After induction, cells were grown for 48 h, stressed or not, and simultaneously treated with 2.5 µM CellROX® Green Reagent for 30 min. Finally, cells were trypsinized and resuspended in 1x PBS.

Green Reagent fluorescence was detected using the 488-nm excitation laser and a 530/30-nm bandpass emission filter in FACS Aria II (BD Biosciences). *A* *total* of 10,000 events were collected using the standard 200 µL/minute collection rate.

## Results

### New stable clone models exhibit 1:1 exogenous-endogenous desmin expression level and low basal aggregation rate

**Figure 1:**
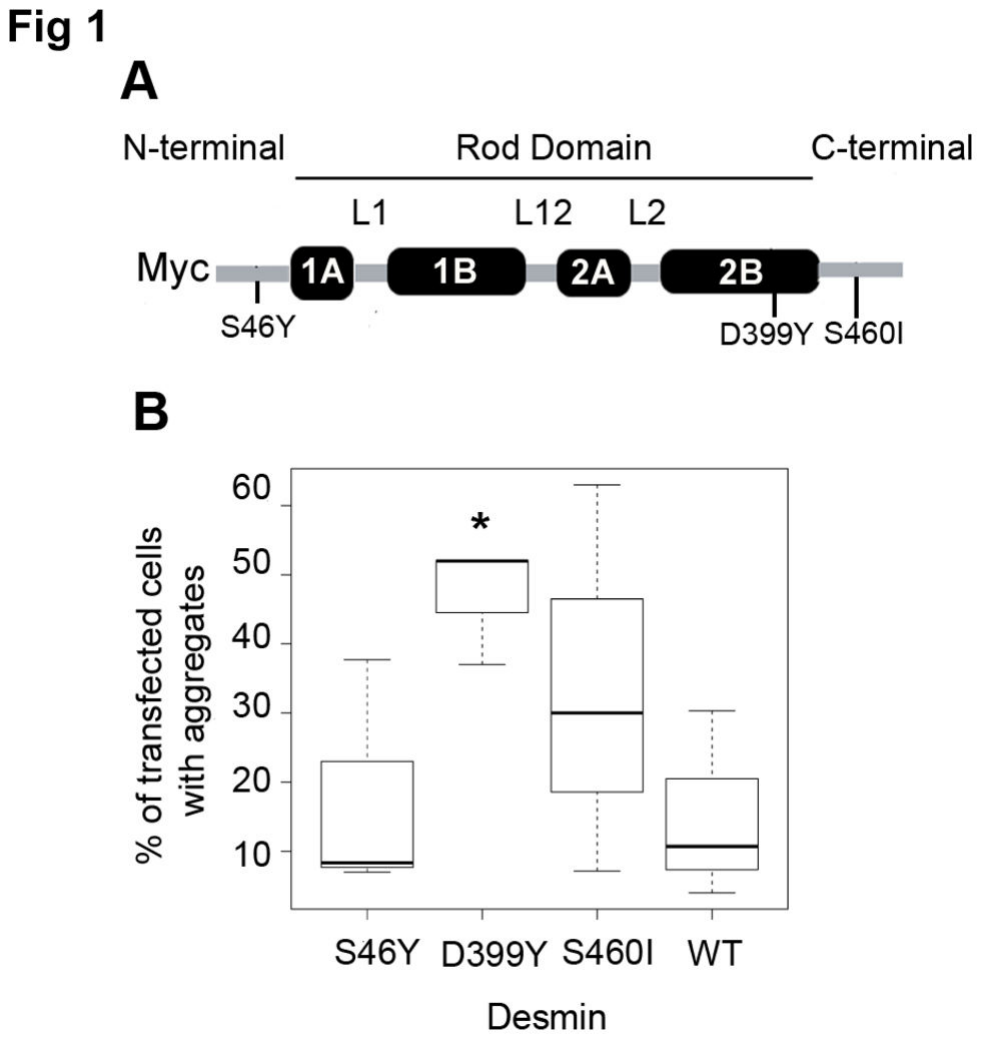
Mutation locations within desmin structure and transient desmin aggregation rates. (**A**) Tripartite desmin representation with S46Y mutation in N-terminal Domain, D399Y in Rod Domain, and S460I in C-terminal Domain. Desmin is Myc-tagged at its N-terminus. (**B**) C2C12 cells transient transfected with plasmids encoding the desmin WT or mutants were stained for immunofluorescence of desmin. Quantification of transfected cells containing aggregates in box plot was performed on three independent experiments. Significant differences are indicated with asterisk (p<0.05) calculated with non-parametric test.

Previously, dominant negative effects of desmin mutants were studied *in cellulo* using transient overexpression experiments in desmin-expressing cells such as C2C12 or BHK21 [9,12]. In the presence of endogenous desmin or vimentin (as in C2C12 cells), mutant behaviour can be arbitrarily separated into three different aggregation state classes: without aggregation (as in DesWT or DesS46Y) [32]; strong aggregation (as in DesD399Y) [12]; and a more variable intermediate phenotype likely dependent on expression level (as in DesS460I) [9] (Figure 1). However, in this context exogenous desmin was more highly expressed than endogenous protein (data not shown).

To obtain a cell model more closely resembling expression in desminopathies, with a mutant/WT desmin expression ratio of around 1:1, we established doxycycline inducible, stable cell clones expressing Myc-tagged desmin (Figure 1A). First, in order to assess that doxycycline does not interfere with aggregation, we transiently transfected C2C12 cells with desmin variants in presence or absence of doxycycline (Data S1). We show that presence of doxycycline has no significant effect on desmin aggregation in C2C12 cells unlike in HSPB5-R120G cardiomyopathy mouse model [33]. Then we selected three of the sixty described desmin mutations, each resulting in different aggregation phenotypes and with mutations in different desmin domains: DesS46Y in head, DesD399Y in rod, and DesS460I in tail (Figure 1A). DesD399Y and DesS460I mutations result in both cardiac pathology and skeletal muscle weakness [9,34]. The DesS46Y phenotype remains unclear [13], but *in cellulo* desmin networks can be formed in 3T3, HL1 [32], or C2C12 cells (Figure 1B). In addition, DesS460I and DesS46Y substitutions affect residues that could be potential phosphorylation sites.

**Figure 2:**
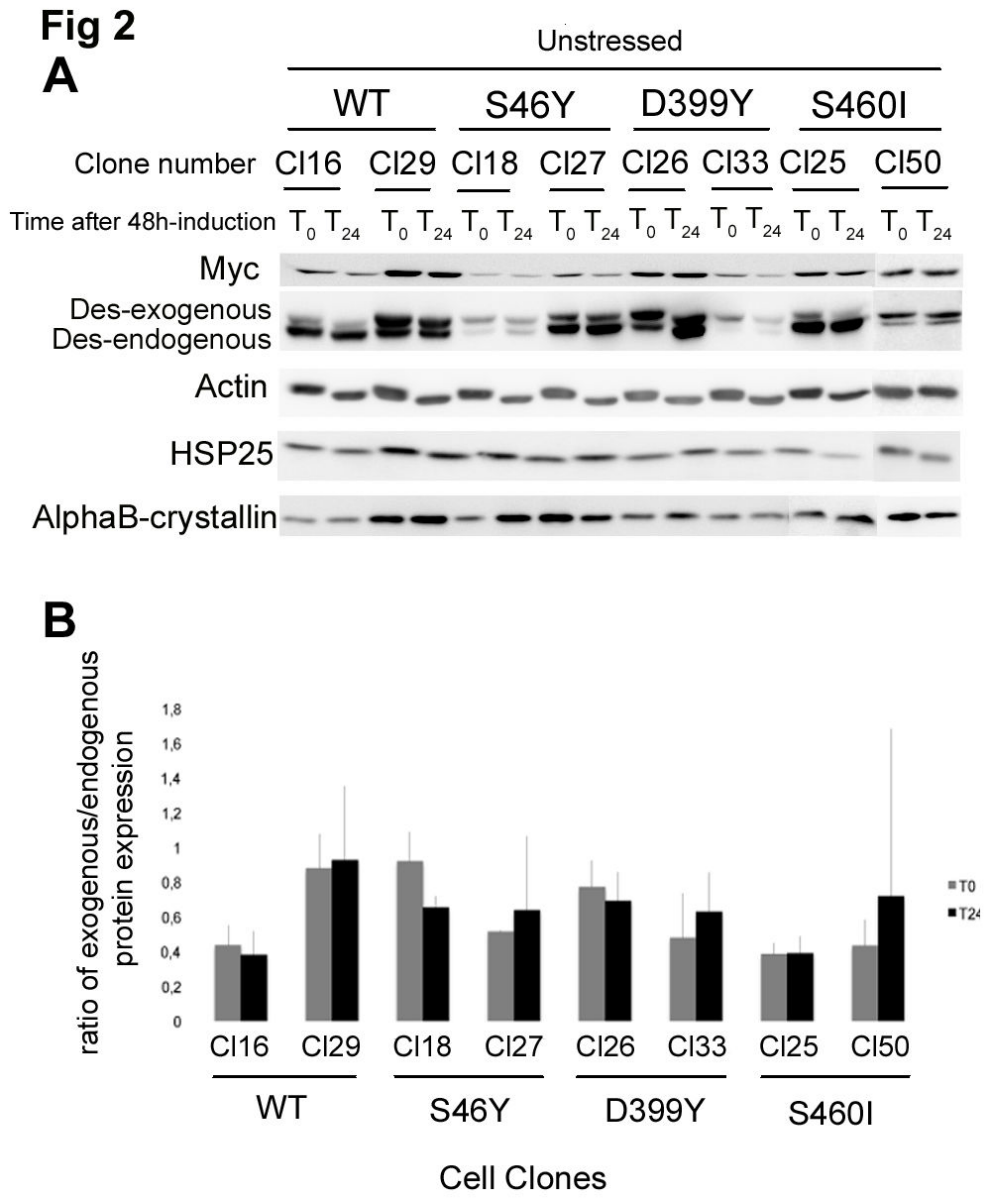
Stress experimental procedure and steady state desmin or sHSPs expression levels. (**A**) Western blot analysis of four endogenous proteins and human desmin in inducible stable cell lines 48 h (T_0_) or 72 h (T_24_) after induction. Equal amounts of proteins probed with anti-alpha-actin antibody to normalize protein levels. (**B**) Quantification of exogenous vs endogenous desmin ratio with ImageJ software. “Cl-Number” represents the name of the selected clone for each desmin mutation.

**Figure 3:**
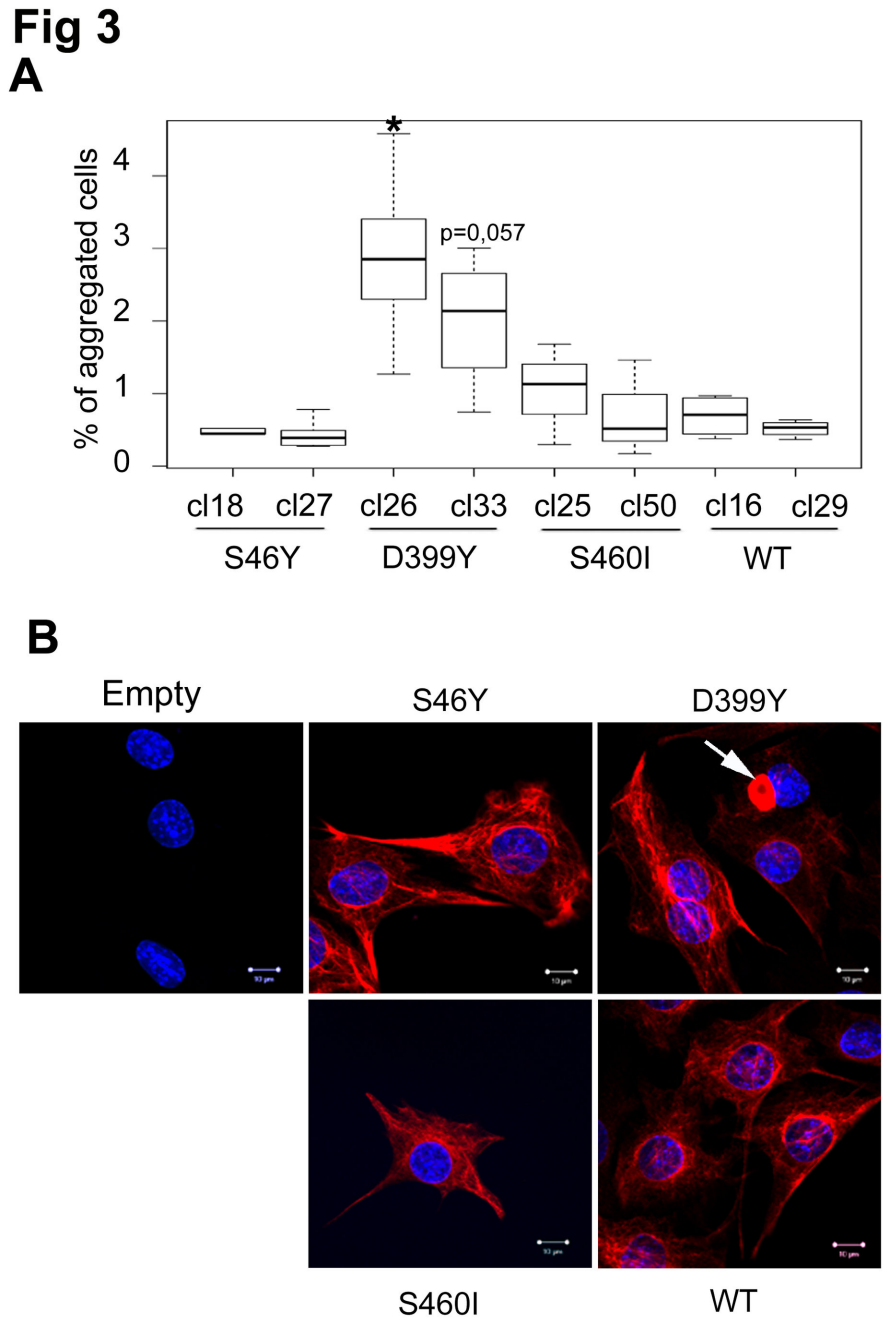
Basal desmin aggregation rates in stably-expressing cells. (**A**) Quantification in box plot of cells containing aggregates for different desmin WT or mutated Tet-On constructs, 48 h after desmin expression induction. Cell counting was performed on three independent experiments (over 100 cells each). Significant differences are indicated with asterisk (p<0.05) calculated with non-parametric test. “Cl-Number” represents the name of the selected clone for each desmin mutation. (**B**) Immunostaining: in red, Myc-desmin; in blue, Hoechst staining. Only DesD399Y-expressing cells form large aggregates throughout the cytoplasm and near nucleus (white arrow). White bar =10µm.

Western blotting was used to detect protein expression (Figure 2A). We selected one clone with high expression and one with low expression for each desmin mutation and for WT desmin; one control containing an empty vector was also used (Figure 2A). At steady state, endogenous desmin was always more highly expressed than the exogenous protein (max. 1:1 ratio for DesWT clone 29), meeting our aim of producing a model system more representative of patient samples (Figure 2B). We investigated expression of alphaB-crystallin, a small heat-shock protein (sHSP) that is known to be a desmin partner and is associated with MFM pathologies. AlphaB-crystallin and HSP25 expression was not upregulated in desmin mutants (Figure 2A), suggesting that desmin mutation does not induce these sHSP responses consistent with previously described patient data [35]. Interestingly, basal aggregation was low, <1.5%, for 3 of four cell lines: DesWT, DesS46Y, and DesS460I (Figure 3A). The DesD399Y line exhibited cells with a large aggregate near the nucleus, which was sometimes misshapen (Figure 3B, white arrow). Aggregation rates were between 1 and 5% in this line, suggesting that DesD399Y remains more aggregative than other mutants. The relatively low rate of aggregation may, however, represent an early stage of pathology.

### Stresses induce a high aggregation rate in DesD399Y

**Figure 4:**
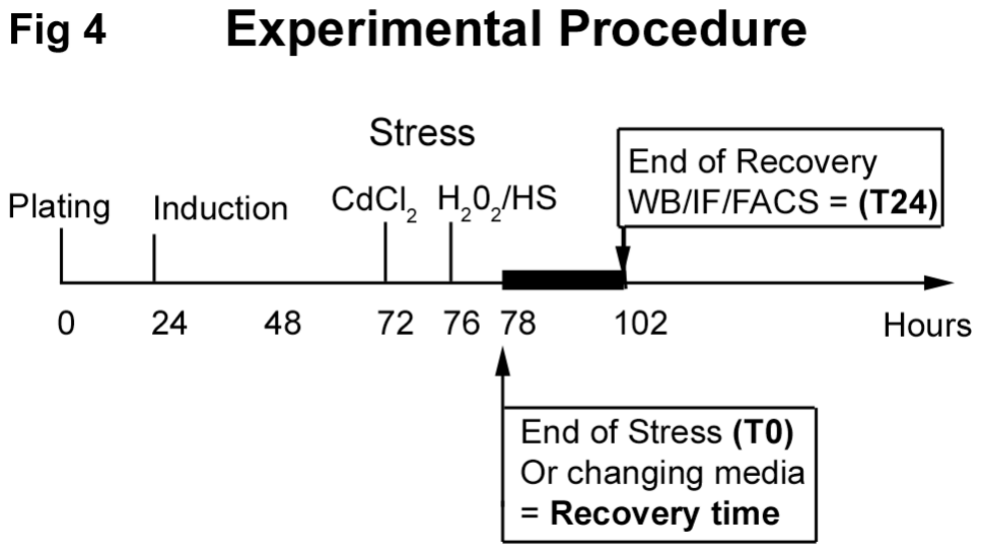
Experimental procedure. Cells were seeded at 3 x 10^3^ cells/cm^2^, desmin expression was induced with doxycycline 24 h later. Stresses were introduced 48 h after desmin induction as described in material and methods; untreated cells were manipulated at the same time. Results were monitored immediately after (T_0_) or after 24 h of recovery time with changing media (T_24_).

**Figure 5:**
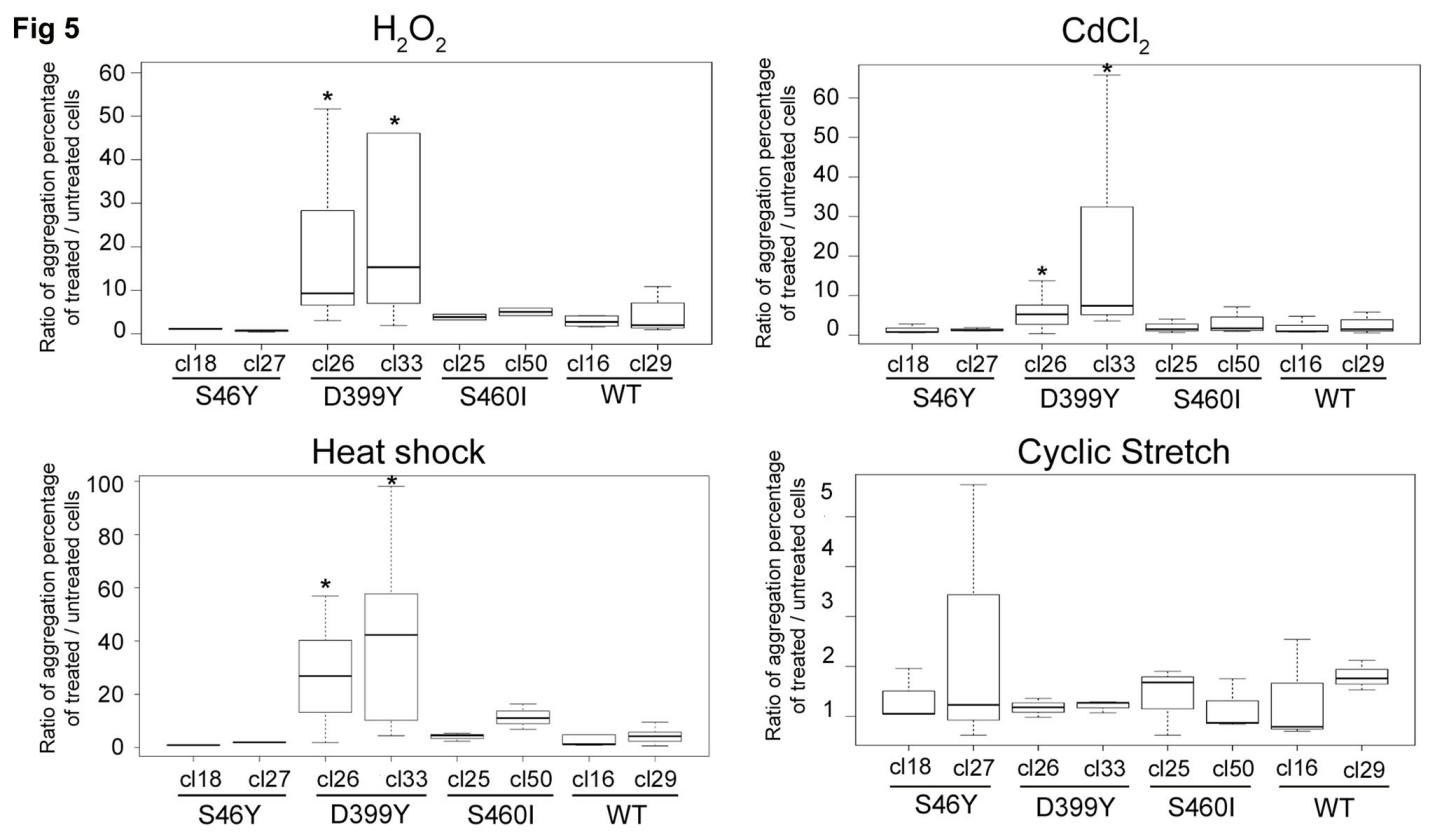
Stress experiments results. Quantification in box plot of cells containing aggregates 24 h after stress (T_24_) in different desmin WT or mutated Tet-On constructs. Four different treatments are shown: oxidative (H_2_O_2_ & CdCl_2_), thermal (heat shock), and mechanical (cyclic stretch). Cell counting was performed on at least three independent experiments (over 100 cells each). Significant differences to both WT clones are indicated with asterisk (p<0.05) calculated with non-parametric test. “Cl-Number” represents the name of the selected clone for each desmin mutation.

To determine the influence of environmental factors on aggregate formation, we perturbed cellular homeostasis with external stresses (chemical, thermal, or mechanical according to the experimental procedure reported Figure 4. Regardless of the stress or mutation studied, a slight increase in aggregation rates occurred just after stress (T_0_) (data not shown). However, after 24 h of recovery, significantly different aggregation patterns were observed (Figure 5): DesWT, DesS46Y, and DesS460I-expressing cells returned to normal patterns 24 h after stress induction. In contrast, DesD399Y-expressing cells exhibited a significant increase in the aggregation rate (between 5 and 40 fold), but only after heat or chemical stresses. Surprisingly, mechanical stress did not affect aggregate formation in this line (Figure 5, right down panel). DesD399Y therefore seems to be more sensitive to external stress, making it an interesting cell model for induced aggregation.

### Stress-induced aggregation has no effect on cytoskeletal or nucleoskeletal organisation and is independent of endoplasmic reticulum stress

Because heat and chemical stresses resulted in increased desmin aggregation in DesD399Y-expressing cells, we investigated whether aggregation near the nucleus resulted in alterations in other networks, particularly cytoplasmic or nuclear architecture. We investigated alpha-actin and alpha-tubulin organization and nuclear intermediate filaments A/C and B lamins. Desmin overexpression did not affect the structure of these networks at steady state (Data S2). Under stress conditions, the presence of large aggregates did not disrupt the networks and no co-localization was detectable (Data S3, S4 and S5).

Massive stress-induced aggregation in DesD399Y-expressing cells may be associated with endoplasmic reticulum (ER) stress. To investigate this hypothesis, we monitored expression of calnexin and BIP, respectively an ER membrane marker and two major ER chaperons and CHOP, a regulator of ER-stress induced apoptosis. Calnexin did not co-localize with desmin after H_2_O_2_ stress, even in the presence of large aggregates (Data S6A). BIP another ER chaperon did also not increase after stress (Data S6B). CHOP expression was slightly transitory just after CdCl_2_ stress (T_0_), independent of desmin mutation expression (Data S6C). Moreover no significant cell death was detected in all clones after HS or CdCl_2_ stress (Data S7). For H_2_O_2_ an increase (about 10% except for WT-16 and D399Y respectively 50% and 30%) is observed consistent with mild stress in literature. However cell death did not seem to be related to specific DesD399Y aggregation (Data S7). Taken together, these results indicate that ER stress is not induced in desmin mutant cells.

### NAC can protect desmin against stress-induced aggregation

The sensitivity of DesD399Y to exogenous stress prompted us to consider it a useful model for testing potential anti-aggregative compounds that might help prevent stress-induced aggregation. We selected several compounds for testing: dexamethasone, fisetin, and N-acetyl-L-cysteine (NAC).

**Figure 6:**
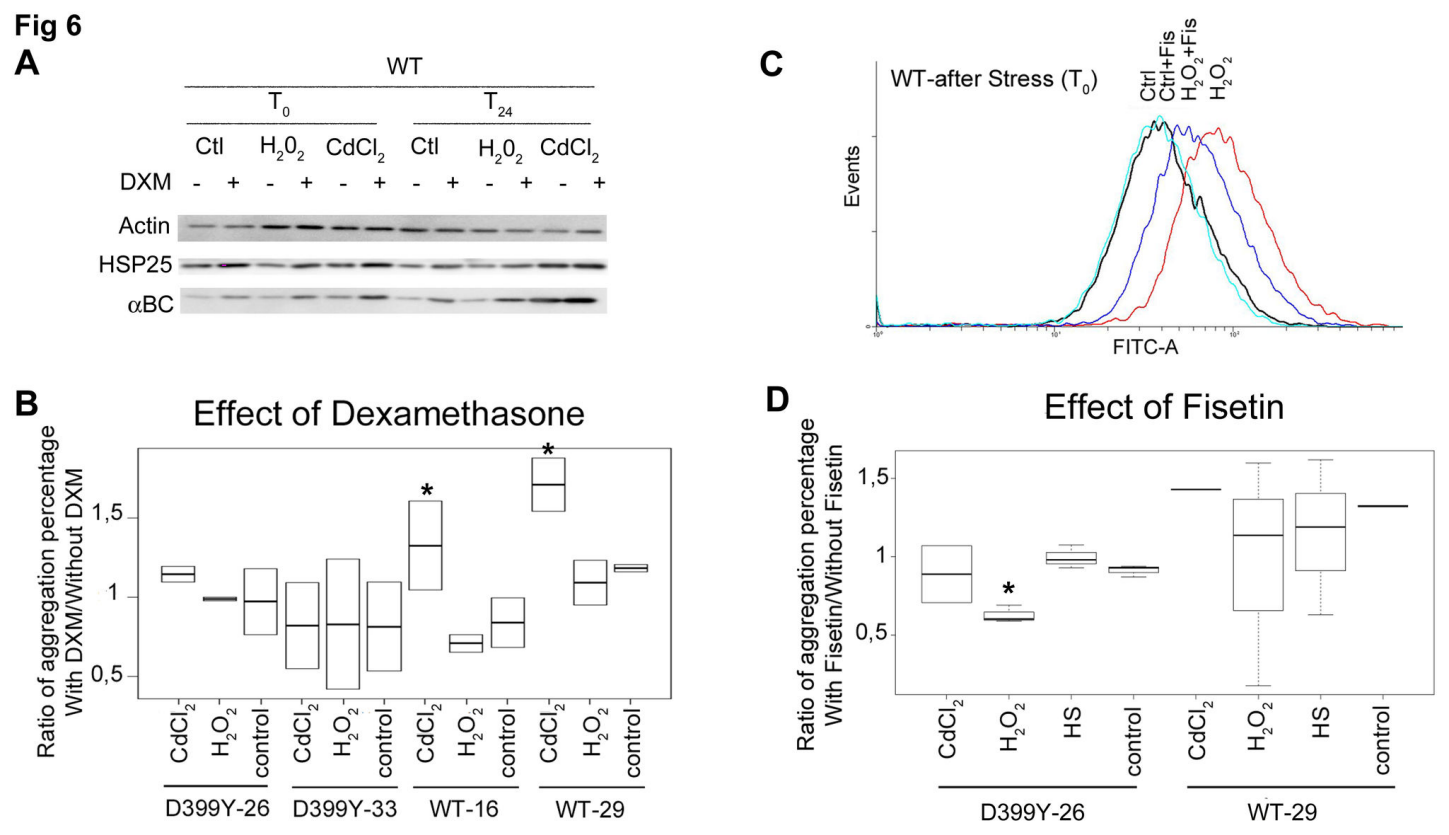
Effect of putative anti-aggregative molecules for reducing stress in our cell model. (**A**) Western blot quantification of sHSP and actin in our various cell lines, unstressed (control) and stressed (H_2_O_2_ or CdCl_2_) after treatment or 24 h after recovery. Equal amounts of proteins probed with anti-alpha-actin antibody to normalize protein levels. (**B**) **and **(**D**) Quantification in box plot of cells containing aggregates, pre-treated 16 h with dexamethasone (2 µM) or fisetin (10 µM). Stressed or unstressed (control) cells were observed after 24-h recovery, and counting was performed on three independent experiments (approx. 100 cells each). For each clone compared to its control, significant differences are indicated with asterisk (p<0.05), calculated with non-parametric test. “D399Y-Number” or “WT-Number” represents the name of the selected clone for each desmin protein. (**C**) Oxidative stress detection using CellROX® Green Reagent fluorogenic probe in live cells just after stress. CellROX® Green Reagent exhibits bright green photostable fluorescence upon oxidation by reactive oxygen species (ROS) measured with flow cytometer. An increase in CellROX® Green Reagent fluorescence intensity (FITC-A in log scale) is observed when cells are treated with H_2_O_2_.

Dexamethasone, a glucocorticoid, induces the sHSP pathway in myocytes [36]. Two members of the sHSP family, HSP27 and alphaB-crystallin (also named HSPB5), have been shown to prevent aggresome formation in the context of alphaB-crystallinopathy (a subgroup of MFM) [37]. We investigated the effect of pre-treating cells with dexamethasone on aggregate formation following stress in cells expressing DesD399Y. HSP25 (mouse equivalent of human HSP27) and alphaB-crystallin were induced by dexamethasone treatment in our stable myoblasts (Figure 6A). However, 24 h after recovery DesD399Y aggregation rates were unchanged. It can be noted a slightly significant increase in the CdCl_2_-exposed WT cells overthrowing dexamethasone protective effect (Figure 6B).

Antioxidants have beneficial effects on various diseases including neurodegenerative pathologies [38,39]. The antioxidant fisetin (3,3’,4’,7-tetrahydroxyflavone) is a bioactive flavonol molecule found in fruits and vegetables that shows promise for improving health [40]. We pre-treated DesD399Y-expressing cells with fisetin and assessed the formation of reactive oxygen species (ROS), pathogenic molecules that are inhibited by antioxidants. Indeed, H_2_O_2_ stress increased ROS levels (Figure 6C). Pre-treatment with fisetin, however, significantly reduced cellular ROS quantity in stressed cells (Figure 6C). However, 24 h after recovery DesD399Y aggregation rates were unchanged, except for a slightly significant decrease in the H_2_O_2_-exposed cells (Figure 6D).

**Figure 7:**
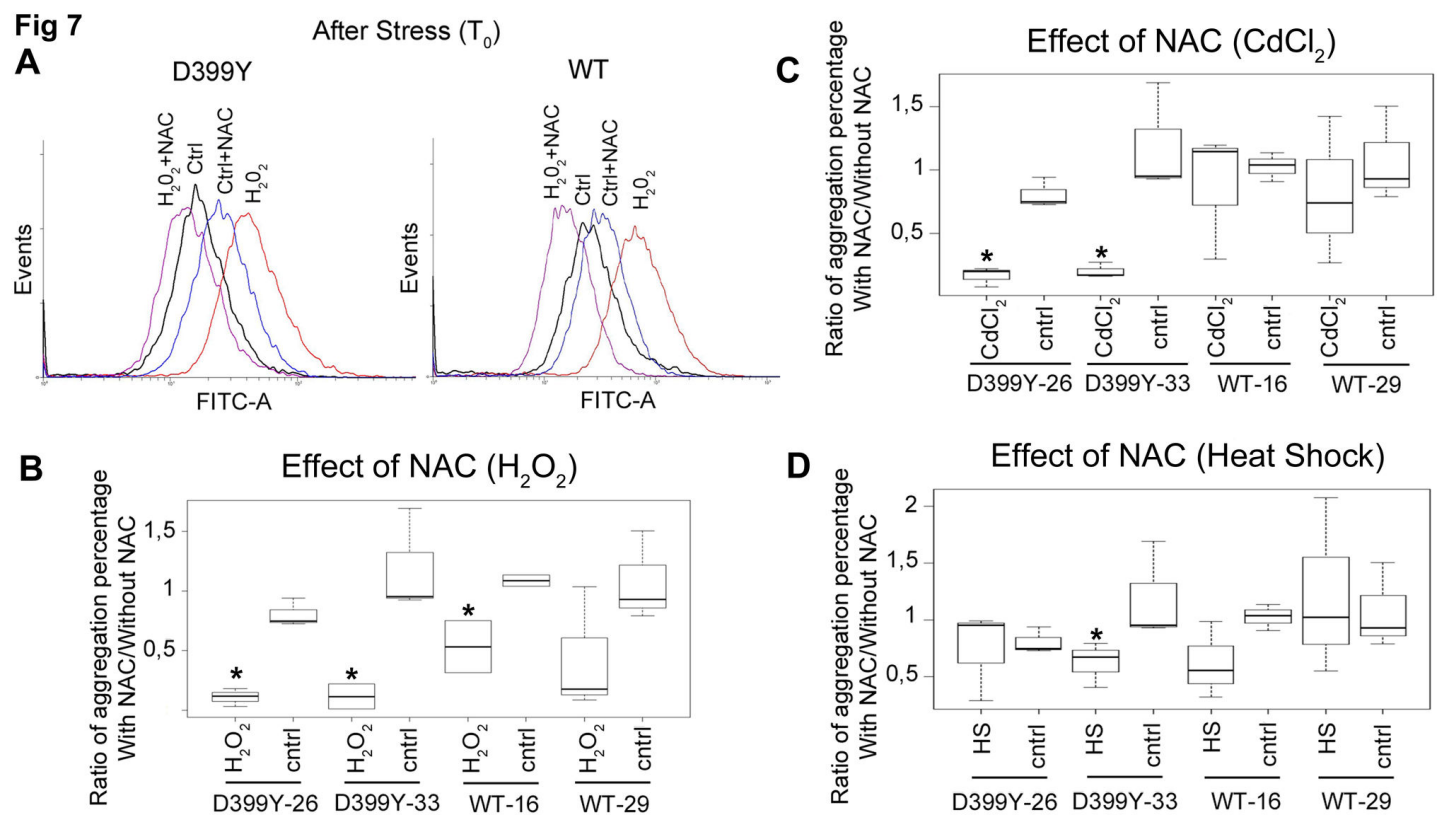
N-acetyl-L-cysteine effect on stress-induced aggregation model. (**A**) Oxidative Stress detection using CellROX® Green Reagent fluorogenic probe in live cells just after stress. CellROX® Green Reagent exhibits bright green photostable fluorescence upon oxidation by reactive oxygen species (ROS) measured in flow cytometer. An increase in CellROX® Green Reagent fluorescence intensity (FITC-A in log scale) is observed when cells are treated with H_2_O_2_. (**B**-**D**) Quantification in box plot representation of cells containing aggregates, pre-treated 16 h before stress with N-acetyl-L-cysteine (10 mM) and counted 24 h after. Cell counting was performed on three independent experiments (approx. 100 cells each). For each clone compared to its control, significant differences are indicated with asterisk (p<0.05), calculated with non-parametric test. Significant differences are indicated with asterisk (p<0.05) calculated with non-parametric test. “D399Y-Number” or “WT-Number” represents the name of the selected clone for each desmin protein.

NAC, another antioxidant, is an approved pharmaceutical drug (Drug Bank accession number: DB06151) used primarily as a mucolytic agent. However, it has recently shown promise in early-onset myopathies resulting from mutations in the *selenoprotein N* gene [38]. As with fisetin treatment, cells pre-treated with NAC did not show an increase in cellular ROS level following H_2_O_2_ exposure (Figure 7A), due to its antioxidant activity. Interestingly, however, 24 h after recovery, DesD399Y aggregation rates were significantly reduced following H_2_O_2_ and CdCl_2_ exposure (Figure 7B, C). Reduced aggregation was not evident after heat shock (Figure 7D), suggesting that different molecular mechanisms lead to protein aggregation following different forms of stress.

## Discussion

In this study, we generated new cell models to compare the stress responses of three desmin mutants and to test potential anti-aggregative molecules. While mouse models and human primary myoblasts have informed our understanding of desmin and desminopathies, models do not exist for all desmin mutants. Moreover, in those contexts, the influence of genetic or environmental factors cannot be easily distinguished. Indeed, the heterogeneous phenotypes within families with desmin mutations underscore the need for identifying contributing genetic or environmental factors.

Our inducible cell lines are derived from an isogenic C2C12 background, allowing for a controlled genetic environment and enabling comparisons between three desmin mutants and four environmental effects. Hydrogen peroxide exposure can be considered as mild oxidative stress resulting in less than 50% cell death (data not shown). Cadmium chloride stress is associated with depletion of the activity of cellular antioxidants like glutathione [41] and up-regulation of stress-inducible proteins [42]. Cadmium intoxication also modifies the expression levels of other important stress-responsive proteins such as HSPs [43].

Surprisingly stress induces only aggregation in cells carrying the DesD399Y mutation. Additionally, aggregation does not occur immediately after stress, but 12 to 24 hours after recovery (Data S8). These results suggest that DesS46Y, DesS460I, and DesD399Y-associated layout mechanisms differ from one to another. This is consistent with results showing specific interactions in aggregates of some desmin mutants (DesE401K, DesR406W, DesE413K) with the IF synemin in contrast to some others (DesD399Y, DesS460I) [44].

Absence of network alterations during and just after heat shock or redox exposure suggests that stress is not directly responsible for aggregation, but that the cellular stress response may be modified. In fact, the presence of the DesD399Y mutation probably disturbs network return to homeostasis after stress, leading to a progressive increase of aggregation (Data S8). Two hypotheses are possible:

-First, stress may depolymerize the desmin network, which then repolymerizes during recovery. With a DesD399Y mutation, repolymerization could be altered. The depolymerization/repolymerization process could occur during mechanical stretch, with desmin re-localization close to nuclei but without aggregation.
-Alternatively, the desmin network might be maintained in a steady state through associated proteins such as alphaB-crystallin. When stress occurs, these specific partners are recruited to protect other essential proteins such as actin [45] or tubulin and, as a consequence, their networks are preserved as shown in data S3 and S4. DesS46Y or DesS460I mutants could remain unaltered, but DesD399Y might not maintain its network and progressively forms aggregates in an irreversible way. However using shRNAs, directed against alphaB-crystallin and HSP25, alone or together did not increase desmin aggregation in steady state nor under stress (Preliminary data not shown) thus suggesting that this alternative does not suit at least in myoblasts.

Notably, heat shock has drastically larger consequences (40-fold aggregation increase) than redox perturbations (10-15 fold). These data indicate that, despite using different pathways, heat shock and redox stress lead to DesD399Y aggregation. Unexpectedly, mechanical stress did not induce aggregation. Cell stretching is supposed to mimic muscle contraction/shrinkage. Here, stretching conditions of 20% elongation and 6 hours’ duration were based on previous experiments on cell reorientation dynamics [29]. Other authors found increased cell death and substrate detachment with 30% elongation and similar frequency [23]. A 20% elongation is sufficient to visualize dynamic, quick modifications but perhaps it is insufficient to detect aggregation occurring in muscles. Stretching is milder than oxidative stress, despite occurring over a longer period, leaving time for other protective process to take place. However, we cannot exclude the possibility that mechanical stress does not induce aggregation in myoblasts. Therefore, myotubes are of interest for stretching experiments. Importantly, we have already seen that differentiated cells adhere less to PDMS sheets, thus technical adjustments will be necessary to pursue this line of study.

It is possible that stress will affect DesD399Y aggregation similarly in myotubes. For other mutations, stress effects may depend on network protein partners only expressed in a more differentiated state. Our results, however, suggest that some desmin mutants are sensitive to stress even in myoblasts. In fact in our cell lines we show low aggregation rate only for DesD399Y (Figure 2) in steady state and chemical or thermal stress drastically increases it. Our results suggest that stress could be a worsening factor for aggregation. Thus desminopathies appear around 20-30 years and it remains unclear when and how aggregates really appear. Moreover patients with the same mutation present different severity and phenotypes that could be related to stress encountered during their life. In some case, patients who practice sport are also more affected. During this long period patients could be exposed to external stress (fever, oxidative stress due to feeding, contraction…) that may progressively lead to cumulative aggregate apparition and subsequent muscle alteration. In addition, the regeneration process in patients may be affected; in particular, aggregation could occur during satellite cell activation and proliferation.

Some drugs (doxycycline or geranyl-geranyl-acetone) have already been successfully tested on cardiac mice model overexpressing an alphaB-crystallin R120G variant without any primary desmin mutation. Considering the different mechanisms involved in aggregation even between desmin variants, our results obtained with DesD399Y establish this cell line as a useful tool for testing anti-oxidative or anti-aggregative molecules on desmin aggregation.

Indeed, dexamethasone, fisetin, and NAC experiments underscore the potential of our models in this type of screening. We focus first on increasing sHSP, which has been demonstrated as efficient in heart HSPB5-R120G model with geranyl-geranyl acetone treatment [28]; second on the maintain of redox homeostasis with antioxidative molecules. For increase of sHSPs, we have tested dexamethasone, which mainly induces two sHSP highly expressed in heart and skeletal muscle instead of geranyl-geranyl acetone, which induces HSPB8 (HSP22) less expressed in skeletal muscle [46] and subsequent in undifferentiated myoblasts as C2C12.

Surprisingly, despite the fact that dexamethasone induces HSP25/27 and alphaB-crystallin, both known to confer resistance to heat-shock, heavy metals [43], or oxidative stress [47], it does not decrease DesD399Y stress-induced aggregation. In fact, dexamethasone induction of sHSP expression is probably insufficient to activate these proteins, and their activation by phosphorylation is likely required. In fact DesD399Y mutation could alter upstream specific signalling leading to sHSP activation. Thus, testing of other specific molecules that activate p38 pathways leading to alphaB-crystallin phosphorylation [48] may advance these findings.

We also demonstrated that not all antioxidants exert equivalent effects. Fisetin, a diet-derived antioxidant known to reduce ROS species in cases of UV damage, is less effective than NAC for alleviating H_2_O_2_-induced stress. NAC drastically reduces DesD399Y stress-induced aggregation for redox metabolism alteration, but not for heat-shock, probably because it is remotely related to cellular ROS. Other, more efficient chemical compounds may be investigated using our adapted cell model. Indeed, the cell culture models presented here offer an appropriate system in which to test such molecules, prior to use in animal models or patients.

Redox homeostasis seems to be important in desminopathies, is perturbed in many cases [24,49], and no treatment is available. Our findings indicate that NAC is a good candidate for potential preventive therapy in desminopathy. In fact, NAC pre-treatment has recently been shown to reduce pathological oxidative stress in another myopathies due to selenoprotein N or RYR1 mutations [38,39]. Because NAC is an approved drug, it warrants further investigation in animal models as AAV-desmin-expression model developed in our laboratory [50] to determine its value in delaying the onset of DesD399Y-linked desminopathies.

## Supporting Information

Data S1 **Doxycycline does modify desmin aggregation in transient transfection experiments.** Quantification in box plot representation of transfected cells containing aggregates, in presence or absence of doxycycline (10 µg/mL). Cells were transfected 24h after plating with JetPEI. Doxycycline was simultaneously added to the media and cells were fixed, Myc-immunostained and counted 48h after. Cell counting was performed on three independent experiments (approx. 100 cells each).
(TIF)

Data S2 **Desmin over-expression does not alter actin, A/C lamins, or microtubule networks.** Immunostaining of unstressed cells. In green: actin, A/C lamins, or alpha-tubulin; in red: Myc-tagged desmin; in blue: Hoechst staining. All networks remained unchanged even for DesWT expression. White bar =10µm.
(TIF)

Data S3 **Desmin aggregation does not alter actin networks.** Immunostaining of unstressed and stressed cells 24 h after treatment. In green: actin; in red: Myc-tagged desmin; in blue: Hoechst staining. No co-localization was visible nor were there network perturbations following desmin aggregation. White bar =10µm.
(TIF)

Data S4 **Desmin aggregation does not alter microtubule networks.** Immunostaining of unstressed and stressed cells 24 h after treatment. In green: alpha-tubulin; in red: Myc-tagged desmin; in blue: Hoechst staining. No co-localization was visible nor were there network perturbations following desmin aggregation. White bar =10µm.
(TIF)

Data S5 **Desmin network perturbations do not affect lamina.** Immunostaining of unstressed and stressed cells 24 h after treatment. In green: lamins (A/C or B); in red: Myc-tagged desmin; in blue: Hoechst staining. White bar =10µm.
(TIF)

Data S6 **Desmin network perturbations do not disturb endoplasmic reticulum.** (**A**) Immunostaining of unstressed and stressed (H_2_O_2_) cells 24 h after treatment. In green: Calnexin; in red: Myc-tagged desmin; in blue: Hoechst staining. White bar =10µm.
(**B**) Western blot quantification of ER chaperon protein BIP and actin in WT or DesD399Y cell lines, unstressed and stressed (CdCl_2_) after treatment or 6, 12, 24 h after recovery. Equal amounts of proteins probed with anti-alpha-actin antibody to normalize protein levels.
(**C**) Western blot quantification of pro-apoptotic protein CHOP and actin in our various cell lines, unstressed and stressed (CdCl_2_) after treatment or 24 h after recovery. Equal amounts of proteins probed with anti-alpha-actin antibody to normalize protein levels.
(TIF)

Data S7 **Cell death measurements after stress.** Unstressed or stressed cells were analysed by FACS for iodure-propidium incorporation (IP+ cells) at T_0_ or T_24_. Cell death after stress is not related to specific DesD399Y aggregation.
(TIF)

Data S8 **Aggregation kinetics.** Immunostaining of unstressed and stressed cells 12 or 24 h after treatment. In green, Myc-tagged desmin; in blue, Hoechst staining. At 12 h DesD399Y-expressing cells already formed aggregates throughout the cytoplasm. White bar =10µm.
(TIF)
