# Supplementary figures and images for: N-Acetyl-L-Cysteine Prevents Stress-Induced Desmin Aggregation in Cellular Models of Desminopathy
Source: PLoS One. 2013 Oct 1;8(10):e76361. doi: 10.1371/journal.pone.0076361 (PMC3788106; doi:10.1371/journal.pone.0076361)

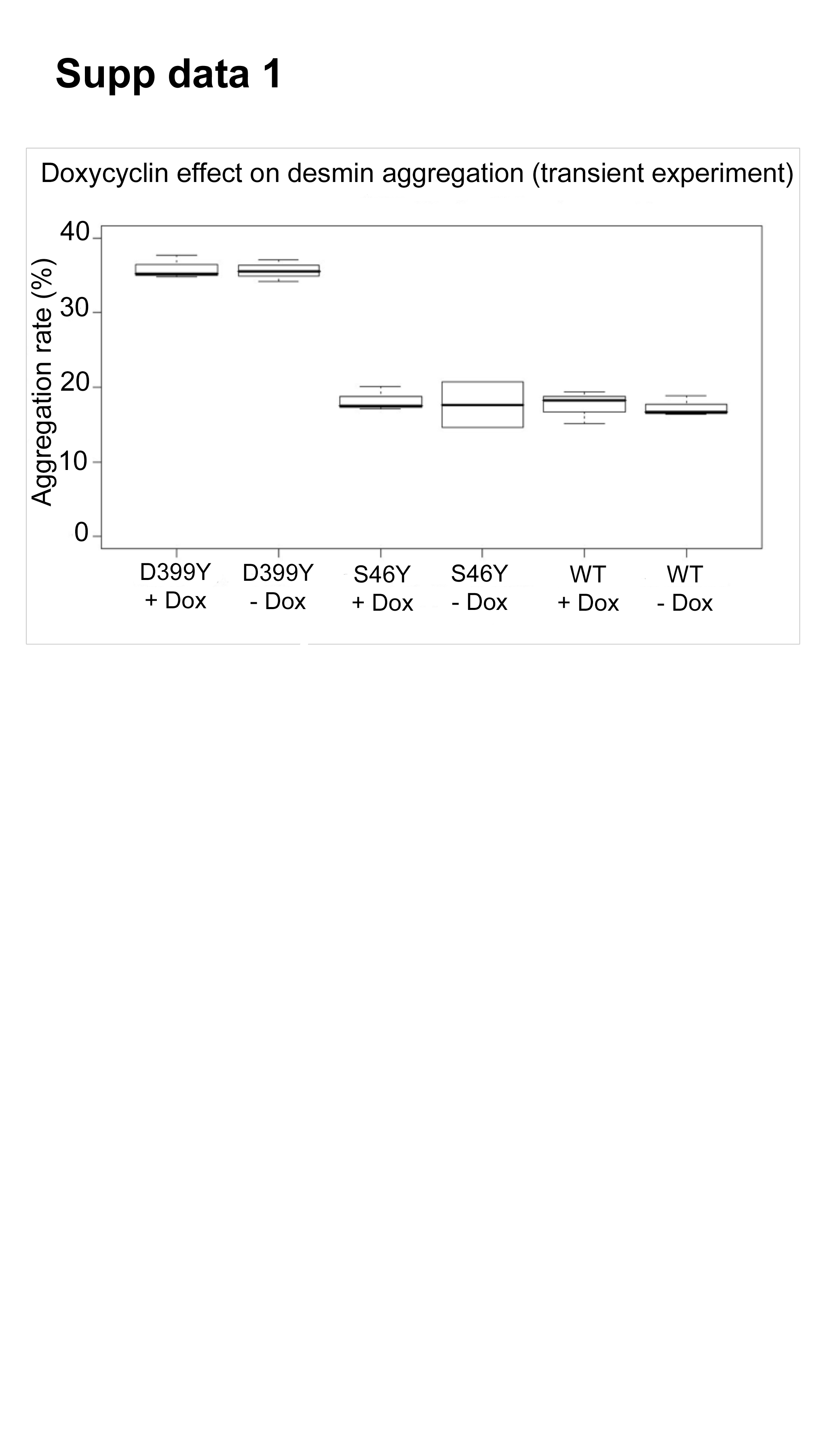

Supplement: Data S1 — Doxycycline does modify desmin aggregation in transient transfection experiments. Quantification in box plot representation of transfected cells containing aggregates, in presence or absence of doxycycline (10 µg/mL). Cells were transfected 24h after plating with JetPEI. Doxycycline was simultaneously added to the media and cells were fixed, Myc-immunostained and counted 48h after. Cell counting was performed on three independent experiments (approx. 100 cells each). (TIF) [file pone.0076361.s001.tif]

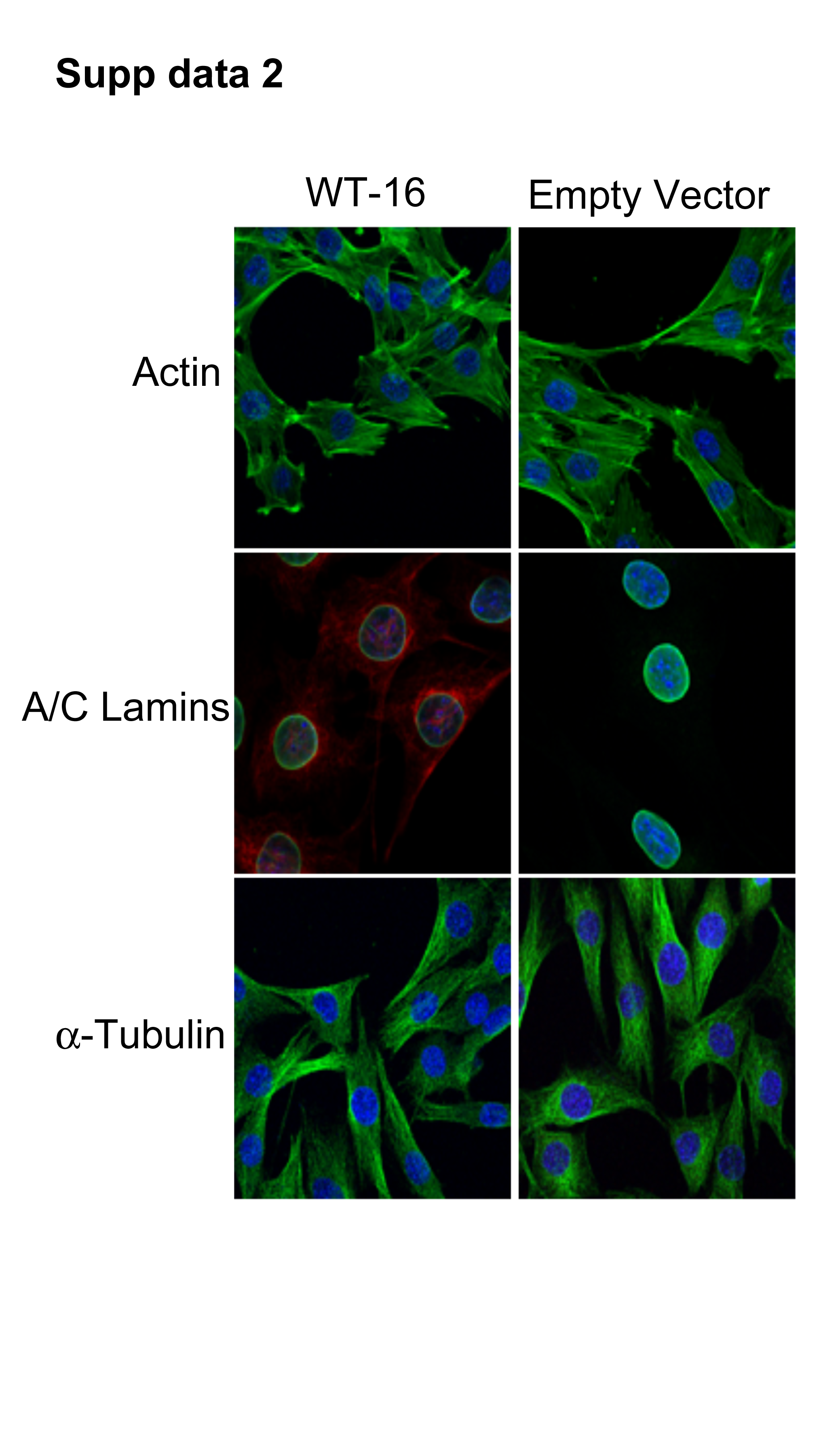

Supplement: Data S2 — Desmin over-expression does not alter actin, A/C lamins, or microtubule networks. Immunostaining of unstressed cells. In green: actin, A/C lamins, or alpha-tubulin; in red: Myc-tagged desmin; in blue: Hoechst staining. All networks remained unchanged even for DesWT expression. White bar =10µm. (TIF) [file pone.0076361.s002.tif]

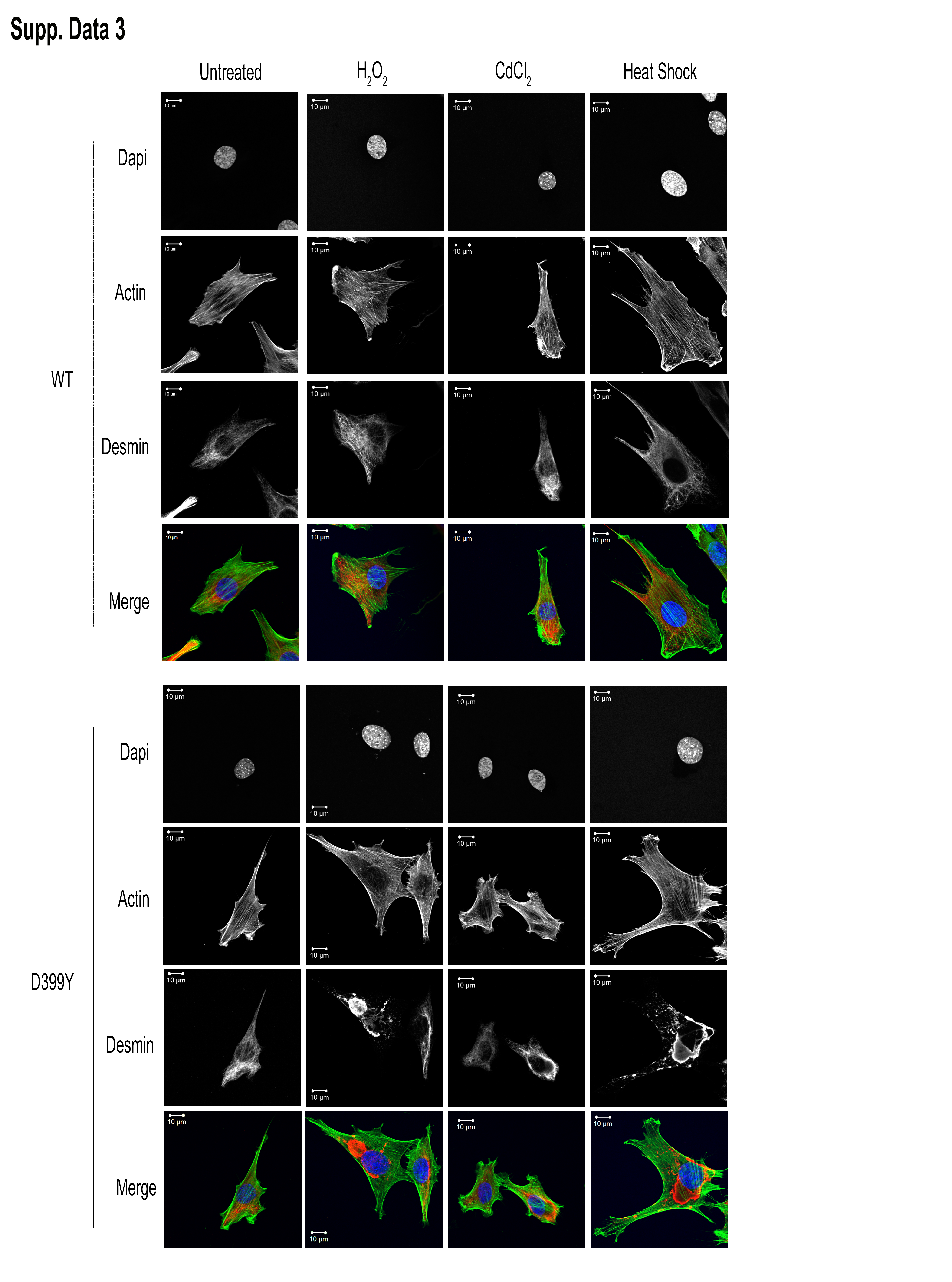

Supplement: Data S3 — Desmin aggregation does not alter actin networks. Immunostaining of unstressed and stressed cells 24 h after treatment. In green: actin; in red: Myc-tagged desmin; in blue: Hoechst staining. No co-localization was visible nor were there network perturbations following desmin aggregation. White bar =10µm. (TIF) [file pone.0076361.s003.tif]

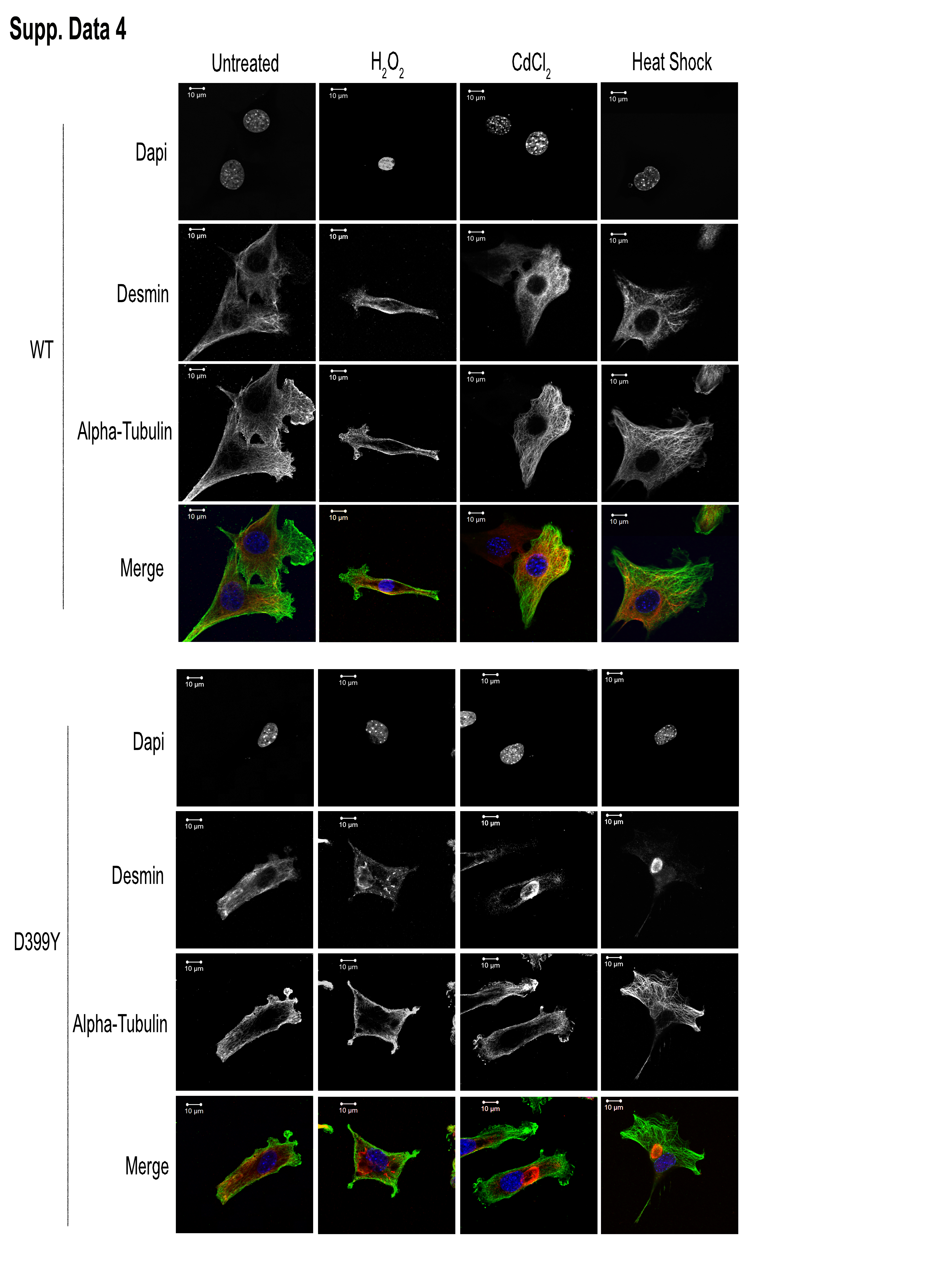

Supplement: Data S4 — Desmin aggregation does not alter microtubule networks. Immunostaining of unstressed and stressed cells 24 h after treatment. In green: alpha-tubulin; in red: Myc-tagged desmin; in blue: Hoechst staining. No co-localization was visible nor were there network perturbations following desmin aggregation. White bar =10µm. (TIF) [file pone.0076361.s004.tif]

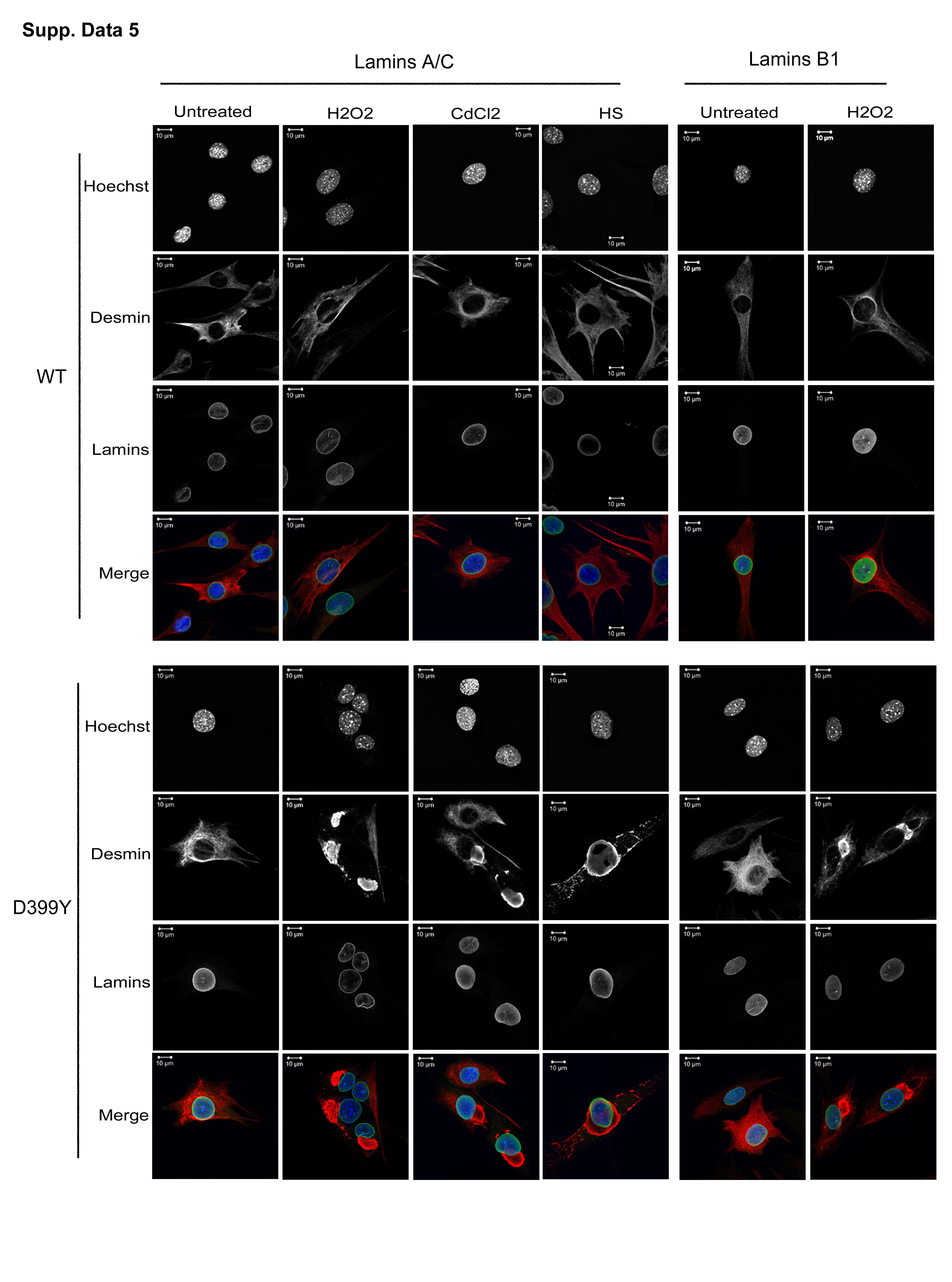

Supplement: Data S5 — Desmin network perturbations do not affect lamina. Immunostaining of unstressed and stressed cells 24 h after treatment. In green: lamins (A/C or B); in red: Myc-tagged desmin; in blue: Hoechst staining. White bar =10µm. (TIF) [file pone.0076361.s005.tif]

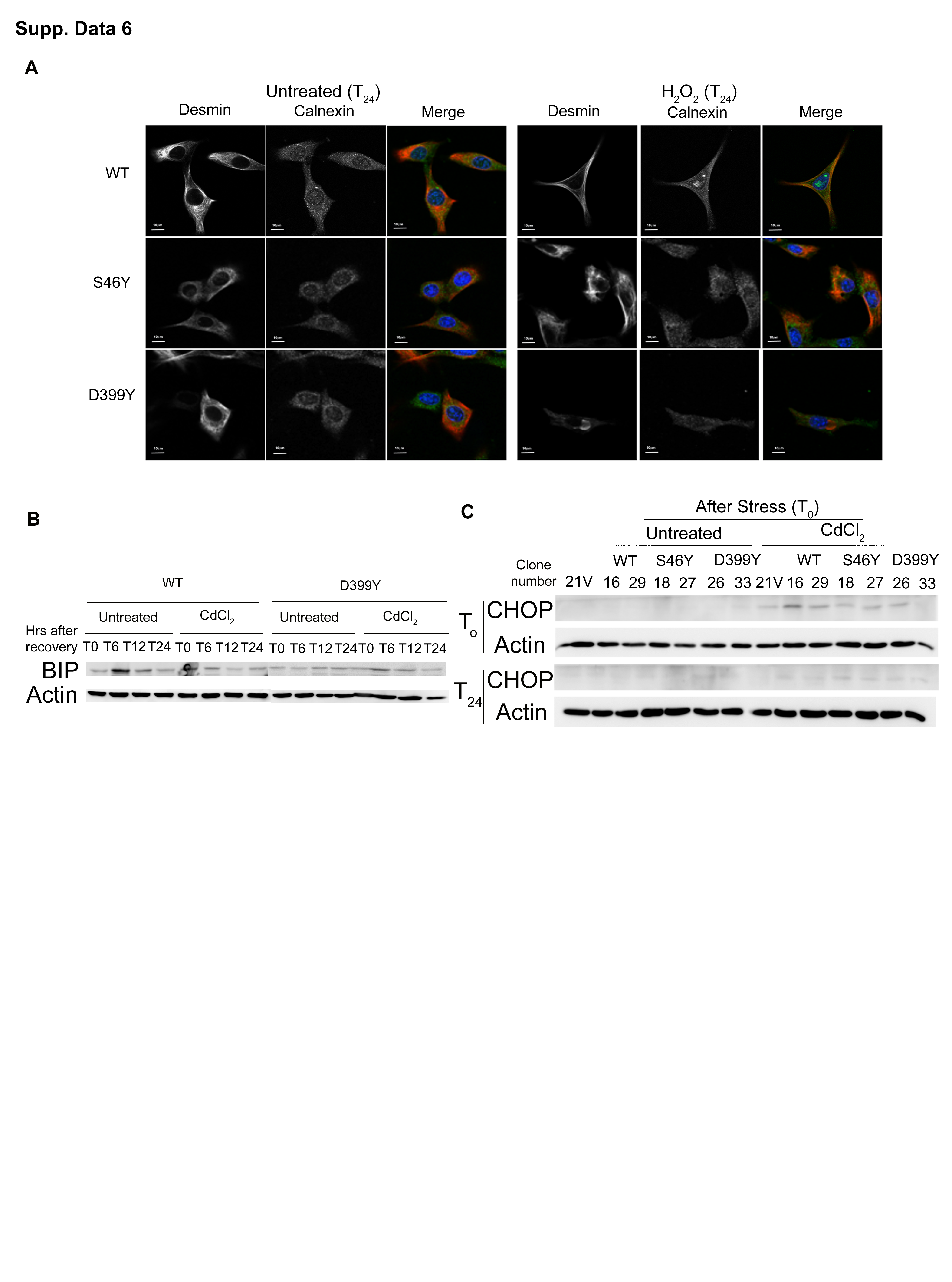

Supplement: Data S6 — Desmin network perturbations do not disturb endoplasmic reticulum. (A) Immunostaining of unstressed and stressed (H2O2) cells 24 h after treatment. In green: Calnexin; in red: Myc-tagged desmin; in blue: Hoechst staining. White bar =10µm. (B) Western blot quantification of ER chaperon protein BIP and actin in WT or DesD399Y cell lines, unstressed and stressed (CdCl2) after treatment or 6, 12, 24 h after recovery. Equal amounts of proteins probed with anti-alpha-actin antibody to normalize protein levels. (C) Western blot quantification of pro-apoptotic protein CHOP and actin in our various cell lines, unstressed and stressed (CdCl2) after treatment or 24 h after recovery. Equal amounts of proteins probed with anti-alpha-actin antibody to normalize protein levels. (TIF) [file pone.0076361.s006.tif]

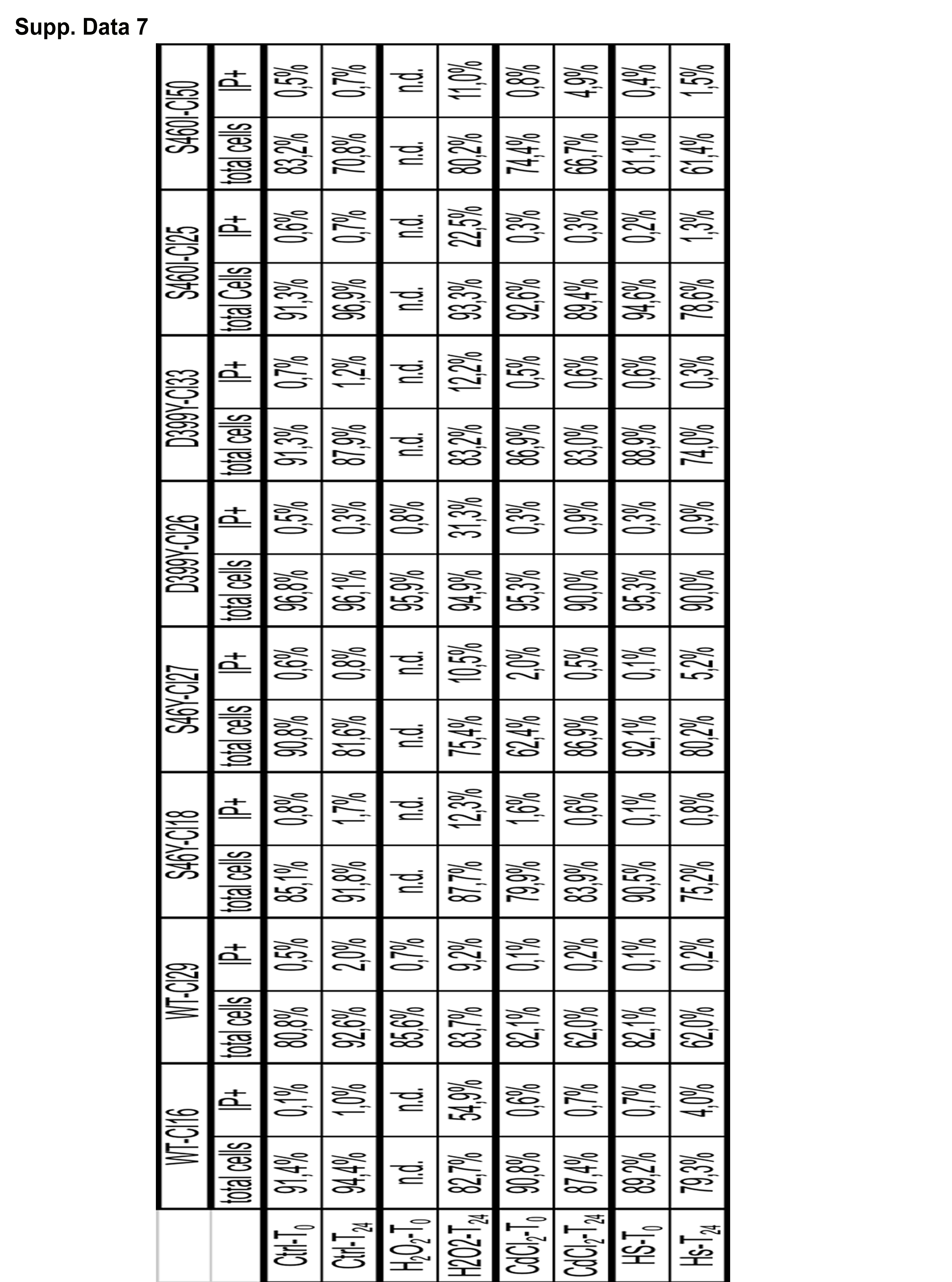

Supplement: Data S7 — Cell death measurements after stress. Unstressed or stressed cells were analysed by FACS for iodure-propidium incorporation (IP+ cells) at T0 or T24. Cell death after stress is not related to specific DesD399Y aggregation. (TIF) [file pone.0076361.s007.tif]

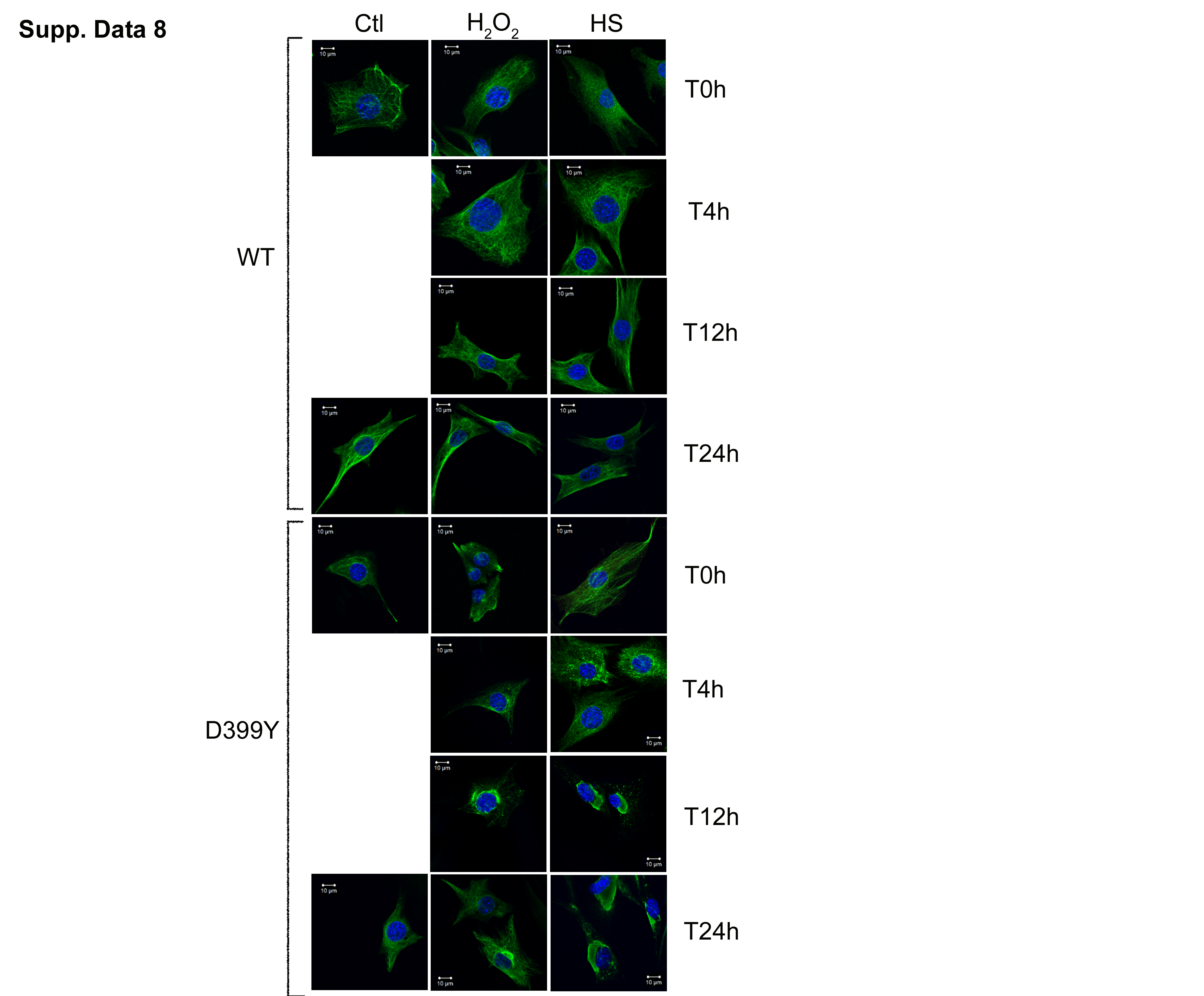

Supplement: Data S8 — Aggregation kinetics. Immunostaining of unstressed and stressed cells 12 or 24 h after treatment. In green, Myc-tagged desmin; in blue, Hoechst staining. At 12 h DesD399Y-expressing cells already formed aggregates throughout the cytoplasm. White bar =10µm. (TIF) [file pone.0076361.s008.tif]
